# Phase II study of SPI-77 (sterically stabilised liposomal cisplatin) in advanced non-small-cell lung cancer

**DOI:** 10.1038/sj.bjc.6603345

**Published:** 2006-09-12

**Authors:** S C White, P Lorigan, G P Margison, J M Margison, F Martin, N Thatcher, H Anderson, M Ranson

**Affiliations:** 1 Christie Hospital NHS Trust, Manchester, UK; 2 Weston Park Hospital, Sheffield, UK; 3 Paterson Institute of Cancer Research, Manchester, UK; 4 ALZA Pharmaceuticals, Mountain View, CA, USA

**Keywords:** NSCLC, SPI-77, liposomes, cisplatin

## Abstract

To determine the efficacy and tolerability of SPI-77 (sterically stabilised liposomal cisplatin) at three dose levels in patients with advanced non-small-cell lung cancer (NSCLC). Patients had Stage IIIB or IV NSCLC and were chemo-naïve, and Eastern Oncology Cooperative Group 0–2. The first cohort received SPI-77 at 100 mg m^−2^, the second 200 mg m^−2^ and the final cohort 260 mg m^−2^. Patients had also pharmacokinetics and analysis of leucocyte platinum (Pt)-DNA adducts performed. Twenty-six patients were treated, with 22 patients being evaluable for response. Only one response occurred at the 200 mg m^−2^ dose level for an overall response rate of 4.5% (7.1% at ⩾200 mg m^−2^). No significant toxicity was noted including nephrotoxicity or ototoxicity aside from two patients with Grade 3 nausea. No routine antiemetics or hydration was used. The pharmacokinetic profile of SPI-77 was typical for a liposomally formulated drug, and the AUC appeared to be proportional to the dose of SPI-77. Plasma Pt levels and leucocyte DNA adduct levels did not appear to rise with successive doses. SPI-77 demonstrates only modest activity in patients with NSCLC.

One of the principal aims of anticancer drug development is to improve efficacy and reduce toxicity through greater tumour specificity. Liposomal formulations of drugs have long held promise as a drug vehicle. Initial difficulties of a rapid clearance of liposomes from the circulation by the macrophages of the reticulo-endothelial system were resolved with the development of sterically stabilised liposomes. Through the addition of methoxy-polyethylene glycol moieties on the lipid bilayer surface, these sterically stabilised (or STEALTH®) liposomes demonstrated prolonged circulation times.

Liposomes have several theoretical benefits that make them attractive as vehicles for anticancer agents. Through encapsulation of the cytotoxic agent, they reduce the potential for side effects resultant from free circulating drug (Ranson *et al*, 1997). Another theoretical advantage of liposomes is their preferential accumulation in the tumour interstitium rather than the microenvironment of healthy tissues. This phenomenon may be due to the passive movement of liposomes through the walls of ‘leaky’ tumour vasculature (Ogihara Umeda *et al*, 1996). This has ultimately translated into the development of liposome-encapsulated cytotoxic agents with demonstrated activity (Ranson *et al*, 1997; Fossa *et al*, 1998).

Cisplatin has been demonstrated to impact positively on survival in advanced non-small-cell lung cancer (NSCLC) (1995), but a downside is substantial systemic toxicity. Liposomal encapsulation of cisplatin has the attraction of potentially reduced toxicity, as well as the potential for tumour targeting. ALZA Pharmaceuticals® (Mountain View, CA, USA; formerly SEQUUS Pharmaceuticals) developed a STEALTH® (sterically stabilised) liposomal formulation of cisplatin (SPI-77) that showed promising results in preclinical models, with significantly greater tumour growth delay than cisplatin. In subsequent phase I studies, the dose was escalated to 320 mg m^−2^ with the dose-limiting toxicity (DLT) of severe proximal myopathy (Meerum Terwogt *et al*, 2002).

In this study, chemo-naive patients with advanced NSCLC were treated with SPI-77 at different dose levels, with the primary end points of response and toxicity, and the secondary end points of survival.

## MATERIALS AND METHODS

### Patient selection

Eligible patients were required to have histologically or cytologically documented Stage IIIB or IV NSCLC deemed not amenable to therapy with curative intent. Patients were required to be chemo-naive, have measurable disease, Eastern Oncology Cooperative Group (ECOG) performance status of 0–2, 24 h creatinine clearance ⩾50 ml min^−1^, adequate bone marrow reserve (Hb⩾10.0 g dl^−1^, neutrophils⩾1500 mm^−3^, platelets⩾100 000 mm^−3^) and adequate hepatic function (serum bilirubin ⩽34 *μ*mol l^−1^, transaminases ⩽2 times ULN). Specific exclusion criteria included clinically significant neuropathy or hearing loss.

All patients gave informed, written consent and the trial was approved by the local research ethics committee and conducted in accordance with good clinical practice obligations.

### Treatment schedule

The first cohort of patients received SPI-77 at a dose of 100 mg m^−2^ every 3 weeks. The second group received SPI-77 at 200 mg m^−2^ and the third group received 260 mg m^−2^. The initial dose level was chosen as an acceptable standard dose of cisplatin, and the final dose level was based on the maximum tolerated dose in the phase I study. SPI77 was administered as an intravenous (i.v.) infusion over 1 h. No routine premedication or antiemesis was given. No additional hydration or diuresis was given. Patients received up to six cycles of treatment in the absence of unacceptable toxicity or disease progression.

### Assessment of response and toxicity

At baseline, patients were assessed with a physical examination (including a meticulous neurological examination), an audiogram and assessment of renal function with calculated or measured 24 h creatinine clearance, and a ^51^Cr-EDTA isotopic GFR scan. Serum chemistry and electrolytes, liver function tests, lipid profile and full blood count was tested.

At cycle completion, reassessment included measured 24 h creatinine clearance, serum chemistry, full blood count and urinalysis. At treatment completion, patients were additionally assessed with audiometry and isotopic GFR scan.

Disease status was assessed at the beginning of treatment and after every other cycle, with either radiological examination (chest radiograph, computer tomography scan) or physical examination. Response was assessed according to standard World Health Organisation (WHO) criteria. Toxicities were graded according to National Cancer Institute (NCI) common toxicity criteria (CTC version 2).

### Statistical analysis

Patients who met all eligibility criteria and received ⩾2 cycles were included in response assessment. Fifteen patients were planned to enter the study, with consideration to recruiting 10 extra patients if one or more responses occurred. The primary study end points were response rate and toxicity, with the secondary end points of overall survival and time to progression. Time to progression was taken from date of first treatment to date of progression. Survival was calculated from date of first treatment to death.

### Pharmacokinetic analysis

Pharmacokinetic sampling was performed predosing, at the end of the 2 h infusion, 1, 3, 5, 24, 48, 72, 96 h postinfusion completion and on days 8, 15 of cycle 1. During subsequent cycles, samples were collected predosing, at infusion completion and at days 8, 15 and 22. At each blood sampling, 5 ml of whole blood was drawn into a heparinised tube. The blood was centrifuged for 10 min at 4°C. Plasma (800 *μ*l) was removed and added to 7.2 ml of 0.1% Triton X-100 containing 0.2% HNO_3_. The samples were divided into eight 1.0 ml aliquots and stored at −20°C. The remaining plasma was ultrafiltered using an Amicon Centrifree ultrafiltration device by centrifugation at 1500 **g** for approximately 30 min using a fixed angle rotor. The ultrafiltrate was stored at −20°C.

Data analysis was performed using ABBOTTBASE® pharmacokinetic systems software.

Each patient's data were analysed by nonlinear least squares regression to determine the individual pharmacokinetic parameters. This utilises iterative nonlinear regression analysis to determine a set of pharmacokinetic parameters that measures *ϕ*, a target objective function. Data from all patients was best fit by a 1-compartment linear model.

AUC extrapolated to infinity (AUC 0–*∞*) was calculated as the sum of AUC (0–*t*) and *C*_pred_/*λz*, where *C*_pred_ is the predicted concentration at time *t*. The apparent terminal elimination rate constant (*λz*) was derived from the log-linear disposition phase of the concentration time curve using least squares regression analysis, with visual inspection of the data to determine the appropriate number of data points to include in the calculation of *λz*. The apparent terminal elimination half-life (*t*_1/2_) was calculated as ln 2/*λz*. The AUC calculations were derived from nonlinear least curves and uniform time/concentration combinations. In eight patients (30.7%) out of 26 patients, data from ultrafiltrates were analysed for free platinum (Pt) and this was expressed as a percentage of total Pt.

### Peripheral blood mononuclear cell preparation

An additional 20 ml of whole blood from study participants was obtained for peripheral blood mononuclear cells.

Two 10 ml blood samples were layered with a pipette onto an equivalent volume of Ficoll® (GE Healthcare, Uppsala, Sweden) in universal tubes. The tubes were centrifuged at 400 **g** for 30 min at 18–20°C. The formed lymphocyte layer was transferred by pipette to another tube, and washed with PBS by centrifugation at 100 **g** for 10 min at 18–20°C. A further wash took place before the cells were stored at −80°C.

### DNA isolation

Tissue samples were thawed to room temperature in a microcentrifuge tube and 600 *μ*l of nuclei lysis buffer (Promega®, Southampton, UK) (12 ml 0.5% EDTA:50 ml of lysis buffer). Proteinase K (Sigma, 17.5 *μ*l of a 20 mg ml^−1^ solution in ddH_2_O) was added and samples were incubated overnight at 55°C with gentle shaking.

After digestion, 200 *μ*l of protein precipitation solution (Promega) was added to the microcentrifuge tube and samples were vortexed for 20 s. Specimens were placed on ice for 5 min and then centrifuged at 15 000 r.p.m. for 5 min at 4°C.

Supernatant (700 *μ*l) from each tube was aspirated and added to 800 *μ*l of isopropanol in a separate tube. Samples were inverted several times until a DNA mass formed. The specimens were centrifuged at 15 000 r.p.m. for 2 min at 4°C. The supernatant was discarded and the DNA pellet was washed with 600 *μ*l of 70% ethanol. Specimens were again centrifuged at 15 000 r.p.m. for 2 min at 4°C. Once more, the supernatant was discarded and the specimens were allowed to air dry for at least 15 min. Samples were subsequently rehydrated with 100 *μ*l of distilled H_2_O. Specimens were incubated at 65°C for 1 h with regular mixing. All samples were then sonicated to ensure homogeneity and stored at −20°C until analysed.

### DNA quantification

Two 10 *μ*l aliquots of the 0, 5, 10, 15, 20, 25, 37.5 and 50 *μ*g ml^−1^ calf thymus DNA (Sigma) standards were placed in wells of a 96-well microtitre plate.

An aliquot of the sample to be analysed was diluted with appropriate volumes of Buffer I and vortex-mixed. Ten microlitres of the diluted sample was placed in each of two wells on the microtitre plate. Hundred microlitres of Hoechst dye solution (9 ml ddH_2_O:1 ml TNE buffer:10 *μ*l Hoechst dye (Hoechst 23358 (Polyscience, Niles, Illinois, USA))) was added to each well. Fluorescence was measured using an FL500 fluorescence microtitre plate reader and DNA concentrations were extrapolated from the standard curve. The DNA concentration of each sample was then corrected for the original dilution. Duplicate values varied by no more than 5%.

### Platinum quantification

Pt analysis was carried out by inductively coupled plasma mass spectrometry (ICP-MS), using a standard Plasma-Quad II instrument (VG Elemental, Winsford, UK), fitted with a standard autosampler and concentric nebuliser, using a method based on that developed by Christodoulou *et al* (1996). Solutions were prepared in 0.1 Mnitric acid (Aristar grade, Merck Scientific, West Drayton, UK), and the assay was calibrated against Pt-containing solutions prepared by serial dilution from a certified reference standard. Measurements were made at the two highest abundance isotopic mass numbers, 194 (32.9%) and 195 (33.8%). The calibration was linear (typical CV <2%) over the analytical working range (0.01–100.0 *μ*g dm^−3^). The Pt-blank reading, obtained from the 0.1 M nitric acid stock solution was subtracted from sample measurements, and in most cases was below 1% of the sample measurement. Procedural blanks were prepared alongside samples to test for inadvertent problems of contamination, but none were detected.

Solutions were diluted gravimetrically in acid-rinsed polyethylene bottles. Blood plasma was generally diluted 20-fold with 0.1 M nitric acid, and for samples with high Pt levels, greater dilution factors were employed as necessary to bring the Pt concentration within the working range. DNA solutions were resuspended in 0.1 M nitric acid (10 ml) before direct aspiration into the ICP-MS. In all cases, uniformity of analytical response was established by the method of additions. The estimated analytical precision was <±10%, except for the DNA samples where the very low Pt concentrations resulted in a lower precision of measurement, estimated at ∼20%.

## RESULTS

### Patient characteristics

Twenty-six patients entered the study at the Christie Hospital, Manchester and Weston Park Hospital, Sheffield between March 1998 and June 1999. Patient characteristics are listed in Table 1. A total of 57.7% of patients were male and the median age was 61.5 years (range 45–76 years). Out of 26 patients, 19 patients (73%) had a performance status of 1, and nine patients (35%) had evidence of metastatic disease.

### Chemotherapy administration

The 26 patients received a total of 100 cycles with a median number of 3.5 cycles. Nine patients received SPI-77 at 100 mg m^−2^ for a total of 31 cycles (median three cycles); 12 patients received treatment at 200 mg m^−2^ for a total of 48 cycles (median four cycles); and five patients received treatment at 260 mg m^−2^ for a total of 21 cycles (median four cycles). Only one patient received a dose delay (of 1 week), owing to a falsely low creatinine clearance result. No patients received a dose reduction.

### Response and survival

Patients who fully met the entry criteria and who received and completed at least two cycles of therapy were evaluable for response and survival according to the protocol. Four patients were not assessable for tumour response. Two patients had Stage IIIA disease, one patient did not have the required histological confirmation, and one patient died of early progression, having received only one cycle.

No objective responses occurred at the 100 mg m^−2^ dose level. One patient achieved a partial response at the 200 mg m^−2^ dose level. The duration of the response was 63 days. No additional responses occurred in the 260 mg m^−2^ group. Two patients experienced a minor response; one patient at 100 mg m^−2^ and another at 200 mg m^−2^. The response rate was 0% (0 patient out of eight evaluable patients) at 100 mg m^−2^ and 10% at 200 mg m^−2^ (one patient out of 10 evaluable patients). There were no further responses at the 260 mg m^−2^ so the response rate was 7.1% at doses of ⩾200 mg (one patient out of 14 evaluable patients).

Median time to progression was 12 weeks. Median overall survival was 23 weeks (range: 3.6–90.4 weeks).

Seven patients (27%) received second-line cisplatin-based combination chemotherapy. One response was seen, four patients had stable disease, and two patients progressed through treatment.

### Toxicity

SPI-77 was extremely well tolerated at the administered schedule. No significant myelosuppression occurred with the maximum toxicity being Grade 2 anaemia in 30% of patients. Three of the 26 patients required at least one blood transfusion (Table 2). Nonhaematological toxicities were generally mild and subjective tolerability was excellent (Table 3). Two patients suffered Grade 3 nausea and vomiting (7.7%). Subsequent nausea was well controlled with the use of 5HT_3_ receptor antagonists. Otherwise, prophylactic antiemetics were not used routinely.

Four patients developed a mild rash (three erythematous maculopapular, one psoriaform). Four patients experienced itch (one episode of Grade 3 itch).

All study patients lacked a history of clinically significant deafness. All patients received baseline audiograms. Out of 26 patients, 15 patients (57.7%) had abnormal audiograms of which 10 patients (67%) out of 15 patients had age-related high frequency loss bilaterally, and five patients (33%) out of 15 patients had moderate-severe sensori-neural loss bilaterally. Sixteen patients had follow-up audiograms although 13 of these were abnormal at baseline. Only one patient out of 16 patients had a significant change, with a 40 decibel (dB) hearing loss at 2000 Hz in one ear, having had moderate to severe high frequency loss at baseline. Another patient was noted to a have a slight deterioration in acuity at 4000–8000 Hz, on a background of moderate high frequency loss at baseline. The median cumulative dose of cisplatin received by the 16 patients was 600 mg m^−2^.

All patients underwent a lower limb assessment of peripheral nerves, testing pinprick sensation, proprioception, vibration and deep tendon reflexes, at baseline and following each cycle. No changes in clinical signs occurred in any patients over the course of treatment.

Patients were assessed pretreatment and at the end of each cycle with either a measured or calculated creatinine clearance. The mean GFR by ^51^Cr-EDTA at baseline for the 26 patients was 80.6 ml min^−1^ (median 90.23 ml min^−1^, range 55–166 ml min^−1^). At the end of treatment, out of 26 patients, 17 patients (65.4%) also had a follow-up ^51^Cr-EDTA isotopic GFR scan. The mean % change in GFR following treatment was −2.16% (median +1.3%, range −27.4 to +20%). The remainder of the group was assessed with a measured or calculated creatinine clearance, with no significant change seen from baseline.

### Pharmacokinetics

One of the two treating centres participated in pharmacokinetic analysis of SPI-77, with data being obtained from 17 patients. A total of 710 samples from 355 time points were collected and analysed from the 17 patients. Nine patients were evaluated at the 100 mg m^−2^ dose level, six patients at 200 mg m^−2^ and two patients at 260 mg m^−2^.

**Figure 1:**
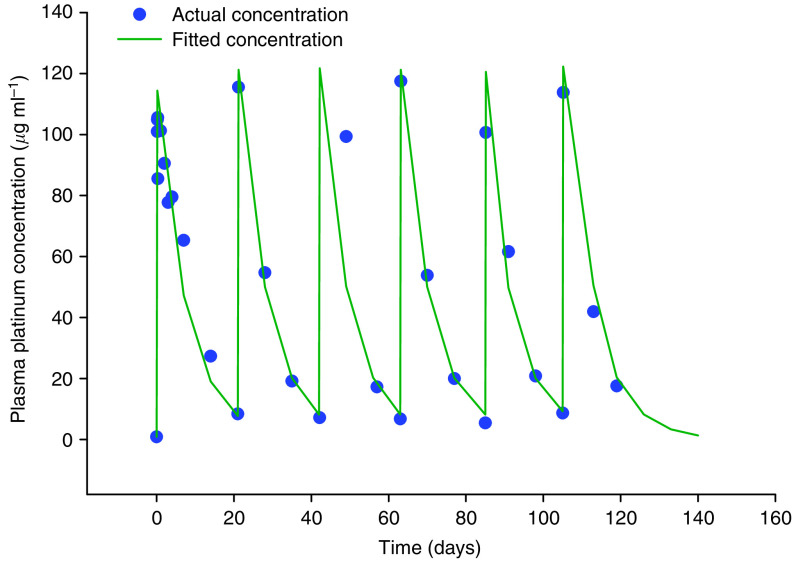
Plasma Pt concentration over time in patient 217, receiving SPI-77 200 mg m^−2^ i.v. 3 weekly over six cycles.

The AUC of Pt (considered a reasonable surrogate for cisplatin/SPI-77) over the first cycle (AUC_1-22 days_) at the 100 mg m^−2^ dose level was a mean of 8233±2369 h *μ*g ml^−1^ with a median value of 9046 h *μ*g ml^−1^ and a range of 4333–11 069 h *μ*g ml^−1^. The mean AUC_1-22 days_ at the 200 mg m^−2^ dose level was 19 822±6187 h *μ*g ml^−1^ with a median value of 21 236 h *μ*g ml^−1^ and a range of 10 765–24 139 h *μ*g ml^−1^. The mean and median AUC_1-22 days_ at the 260 mg m^−2^ dose level was 22 729±4014 h *μ*g ml^−1^ and a range of 19 891–25 567 h *μ*g ml^−1^ (Table 3). No significant accumulation of SPI-77 was noted in subsequent cycles and the AUCs for cycles 1 and 2 appeared similar at all dose levels (Figure 1). The mean AUC_1-22 days_s appeared to increase linearly with dose (*r*^2^=0.54).

**Figure 2:**
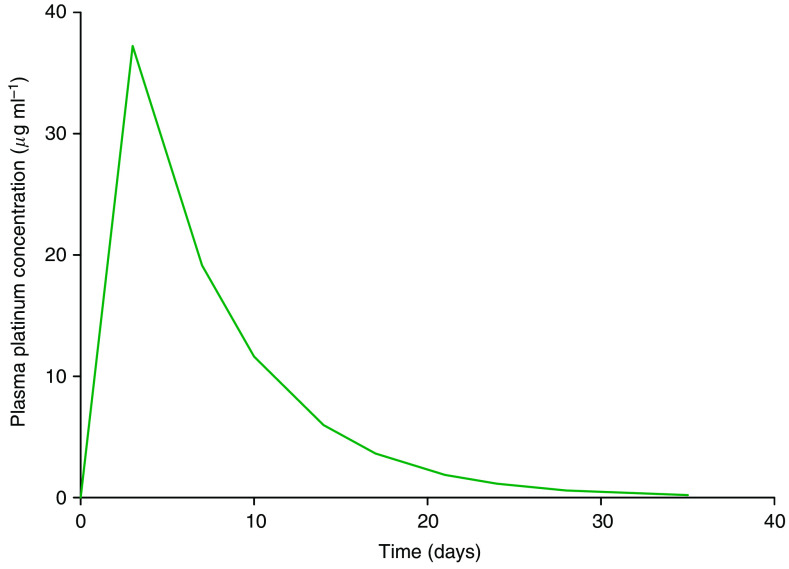
Population concentration over time curve for patients treated with 100 mg m^−2^ SPI-77 during cycle 1.

Drug concentration declined in a mono-exponential way with a mean *t*_1/2_ of 107.4±25.29 h (median 108 h, range 62.3–168 h) (Figure 2). Plasma clearance of SPI-77 had a mean of 0.02181 l h^−1^ (median 0.0188 l h^−1^, range 0.0099–0.0416 l h^−1^) and was independent of dose (Table 3). The apparent volume of distribution (*V*_d_) had a mean of 3.134 l (median 3.24 l, range 2.07–4.08 l) that approximates to plasma volume, and was independent of dose.

Eight patients (30.7%) were assessed for free (ultrafiltrated) Pt levels. These were universally very low, with a mean value of 0.139% of the corresponding total Pt (median 0.048%, range 0.0015–1.1022%). In two patients who were assessed for ultrafiltrated Pt levels over the first two cycles, the *C*_max_ occurred at days 3 and 4 with levels of 0.15–0.16 *μ*g ml^−1^.

**Figure 3:**
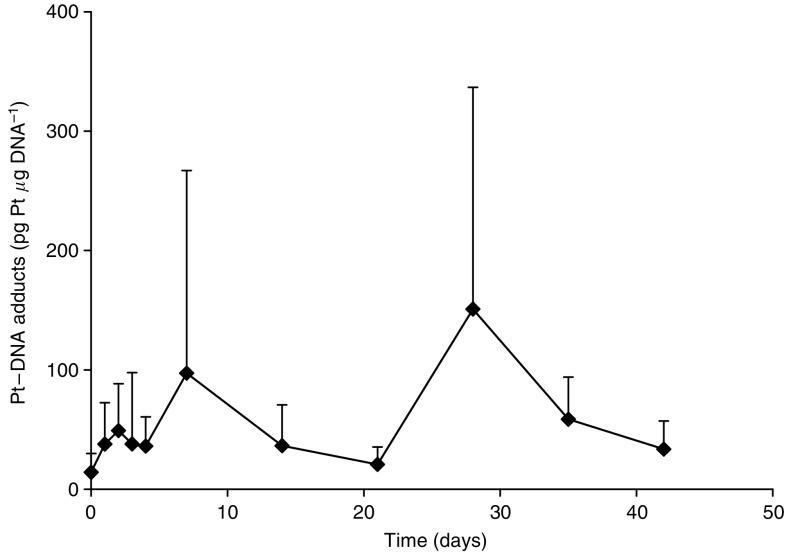
Pt-DNA adducts in patients receiving 100 mg m^−2^ SPI-77 i.v. 3-weekly.

Seventeen patients (65.4%) had extra blood samples taken for lymphocyte collection. Cisplatin-DNA adducts were measured over the course of the first two cycles. At 100 mg m^−2^, the adduct levels peaked at 151±186 pg Pt *μ*g^−1^ DNA on day 4 of cycle 1. A small rise in cisplatin-DNA adduct formation was seen again following the second cycle of SPI-77. The mean levels were lowest at day 1 of the second cycle (18±11 pg Pt *μ*g^−1^ DNA). There was no evidence of accumulation of adducts over time as they returned to pretreatment levels at the end of cycles 1 and 2 (Figure 3). There was no evidence of increased adduct formation when the dose was increased from 200 to 260 mg m^−2^.

## DISCUSSION

The phase II study tested three different doses of SPI-77 to assess activity and toxicity of SPI-77 over an extended number of cycles. Cumulative toxicity is a common clinical problem with conventional cisplatin and has also been a significant problem with STEALTH® liposomal doxorubicin (Ranson *et al*, 1997). A total of 100 cycles of SPI-77 at 100–260 mg m^−2^ were administered to the 26 patients with a median of 3.5 cycles per patient. The experience at higher cumulative doses in the current trial was far greater than the initial phase I study where 21 patients were treated for a total of 59 cycles, at dose levels ranging from 40 to 320 mg m^−2^, at a median number of two cycles per patient (Meerum Terwogt *et al*, 2002).

The objective overall response rate of 4.5% to SPI-77 was disappointing, even for a single agent in NSCLC. No responses were seen at 100 mg m^−2^. If only patients treated at ⩾200 mg m^−2^ were included in response analysis, the response rate was 7.1%. This compares with the activity of single-agent cisplatin in advanced NSCLC of 6–32% in various studies (Thatcher, 1995). The one patient who achieved an objective or partial response progressed by cycle 5. Two other patients had minor responses to SPI-77. The median overall survival of 23 weeks was relatively short compared with the survival of patients treated with single-agent cisplatin in other recent studies (Wozniak *et al*, 1998; Sandler *et al*, 2000). Performance status plays an important role in influencing the duration of longer survival with treatment of advanced NSCLC (Thatcher *et al*, 1995). Given that the patients had relatively good performance status (with PS⩽1 in 73.1%), the disappointing survival in this phase II study would be consistent with SPI-77 having low activity as a single agent in advanced NSCLC.

In contrast to the well-known potential consequences of conventional cisplatin such as nausea, myelosuppression, nephrotoxicity and neurotoxicity, SPI-77 was extremely well tolerated in all patients. Despite the extremely high *C*_max_ and AUC plasma levels of SPI-77, toxicities typical of conventional cisplatin were not seen. This underscores the ability of STEALTH® liposomes to reduce drug toxicity. In this study, myelosuppression was not clinically significant, with no instances of Grade 3/4 or thrombocytopenia. Mild anaemia was common with 31% of patients having Grade 2 anaemia although only three patients required a blood transfusion.

Nonhaematological toxicity was also mild with SPI-77. Grade 3/4 nausea occurred in 7.7% of patients, with no other requirement in the other patients for routine antiemetic treatment such as 5HT_3_ receptor antagonists. This is in contrast to the usual antiemetic protocols required with conventional cisplatin therapy. In the current study, there were instances of hypersensitivity reactions to SPI-77 during the infusion in two patients. Four patients had varying grades of itch, and four patients had a skin rash. None of the patients developed the type or severity of rash that is encountered with STEALTH® liposomal doxorubicin therapy (Ranson *et al*, 1997).

In the case of neurotoxicity, there was no evidence of clinically significant peripheral neuropathy. Additionally, none of the patients showed signs of a proximal myopathy, a feature that was reported in two patients treated at 320 mg m^−2^ in the phase I study of SPI-77 (Meerum Terwogt *et al*, 2002). Only one patient exhibited a change in audiometry with a 20 dB unilateral hearing loss. Although this loss occurred at the typical frequency of normal human speech, the patient concerned denied any tinnitus or subjective change in hearing. In contrast, a study that examined the ototoxicity of conventional cisplatin at doses of 50–100 mg m^−2^ found that deterioration in audiometry post-therapy was frequent (Schaefer *et al*, 1985) with higher frequency loss (4000–8000 Hz) common at cumulative doses of 200 mg. The authors concluded that cisplatin ototoxicity was dose-dependent. Ototoxicity was not consistently seen with SPI-77 despite high cumulative Pt doses (median 600 mg m^−2^, range 200–1560 mg m^−2^), suggesting that cisplatin neurotoxicity was considerably reduced with liposomal encapsulation

Nephrotoxicity was the DLT in the early studies of conventional cisplatin (Finley *et al*, 1985). In a previous study of conventional cisplatin, 15 patients who had received 100 mg m^−2^ every 3 weeks for a median of three cycles had a fall in creatinine clearance from 112 ml min^−1^ at baseline to 65 ml min^−1^ at treatment completion. However, GFR did not change significantly during the course of SPI-77 treatment. Additionally, aggressive hydration schedules are routinely used to reduce the incidence of cisplatin-induced acute renal dysfunction, whereas in this study, patients received no additional hydration or forced diuresis.

The data from the trial reported here have demonstrated that the toxicity profile of SPI-77 was markedly different from conventional cisplatin at either standard doses (75–100 mg m^−2^) or experimental high doses (>100 mg m^−2^). This reduced toxicity owing to liposomal encapsulation has been noted with other liposome-encapsulated cytotoxic agents such as Daunoxome® or Caelyx/Doxil® (Ranson *et al*, 1997; Fossa *et al*, 1998).

In the case of doxorubicin and cisplatin, side effects such as cardiomyopathy (doxorubicin) and nephrotoxicity (cisplatin) have been partly attributed to high levels of peak free drug (Minow *et al*, 1977; Nagai *et al*, 1986). In a study by Nagai *et al* (1986), the plasma *C*_max_ of ultrafiltrated Pt was the most useful pharmacokinetic marker for cisplatin nephrotoxicity. In our study, free cisplatin levels were extremely low in plasma, with a mean value of 0.09 *μ*g ml^−1^. In the two patients who were assessed by determination of serial ultrafiltrated Pt levels, the peak Pt concentration of 0.15–0.16 *μ*g ml^−1^ was 60-fold lower than levels in patients postbolus treatment with 100 mg m^−2^ of conventional cisplatin in a previous study (Vermorken *et al*, 1982). In conclusion, the improved toxicity profile of SPI-77 was likely to be due to stable encapsulation of cisplatin by the liposomes, resulting in minimal free Pt in plasma.

The pharmacokinetics of SPI-77 was assessed in 17 of 26 patients enrolled in the trial. The plasma AUCs of Pt (considered a reasonable surrogate for cisplatin/SPI-77) levels were 100-fold higher than have been reported for conventional cisplatin at 100 mg m^−2^ (Johnsson *et al*, 1996). Plasma clearance of SPI-77 was lower, and *t*_1/2_ longer compared to conventional cisplatin. *V*_d_ in SPI-77 was equivalent to plasma volume, in contrast to cisplatin that has a larger *V*_d_ of 11–24 l (Andersson *et al*, 1996; Johnsson *et al*, 1996). Accordingly, the pharmacokinetic behaviour of SPI-77 follows the typical model of a STEALTH® liposome-encapsulated drug.

The levels of free Pt following SPI-77 were noted to be a minute fraction (0.139%) of the total plasma Pt. It is unclear whether the free cisplatin appears in the plasma as a result of release of drug following degradation of the liposomes by the macrophage–monocyte system or whether the drug becomes bioavailable in plasma following breakdown of the liposomal membrane in the circulation.

Pt-DNA adduct formation was assessed in 17 of the patients with higher levels noted than previously seen in other studies of cisplatin-treated patients. As lymphocyte cisplatin-DNA adducts are presumed to arise from exposure *in vivo* to free cisplatin, lower levels of adducts might have been expected as a consequence of the extremely low plasma free Pt levels post-SPI-77 and this may be a result of carry-over of liposomal Pt during Ficoll separation of lymphocytes and release during later processing. Alternatively, it may be the result of enhanced exposure of lymphocytes to SPI-77 liposomes entrapped in the reticulo-endothelial system sinusoids.

SPI-77 is a novel formulation of cisplatin, encapsulated in sterically stabilised liposomes. Its toxicity in humans is mild, with almost no evidence of Grade 3/4 haematological or nonhaematological toxicity. No significant toxicity peculiar to SPI-77 was noted, with only an occasional mild rash or infusion-related hypersensitivity reaction. This is contrast to palmar-plantar erythema that occurs with Caelyx/Doxil® SPI-77 was easily dose-escalated to 260 mg m^−2^ and total cumulative doses of up to 1560 mg m^−2^ were administered without reaching acute or cumulative DLT.

Toxicity, mainly in the form of liver damage, hampered the execution of tumour xenograft experiments testing SPI-77 (Colbert, 1999). Interestingly, such toxicity was neither demonstrated in larger animals, nor in the present human studies. The interspecies differences in the tolerability of SPI-77 probably reflect differences in the metabolism of the acute lipid-loading inherent with SPI-77. A single published study and unpublished data from our group have demonstrated SPI-77 activity against a variety of tumour xenograft models (Newman *et al*, 1999). Tumour growth delay was significantly longer in tumour xenografts treated with SPI-77 in comparison to conventional cisplatin.

The promising activity of SPI-77 in the animal xenograft studies was not replicated in the clinical trial of patients with advanced NSCLC. In the current phase II study, the response rate was a modest 4.5%. Similarly, no objective tumour responses occurred in the 24 patients in the phase I study (Meerum Terwogt *et al*, 2002), and also in another phase II study in a similar population (Kim *et al*, 2001). Three responses (out of 17 patients) were seen with a combination of SPI-77 and vinorelbine in a phase I study (Vokes *et al*, 2000). In patients with head and neck tumours, it has been administered safely with radiation, but as a single agent was disappointing with no responses (Harrington *et al*, 2001; Rosenthal *et al*, 2002).

The response rate in this phase II study of SPI-77 appears inferior to other known active single agents in NSCLC, including conventional cisplatin, carboplatin and newer agents such as gemcitabine (Thatcher *et al*, 1995). Despite the minimal toxicity of the drug, SPI-77's low activity in advanced NSCLC does not justify further clinical studies in this disease setting.

As significant antitumour activity was demonstrated in the xenograft experiments, the reason for low activity in human studies may relate to host differences or to differences between xenografts and *de novo* cancers in humans. Additionally, the ability to metabolise liposomal drugs is limited in rodents, yet such formulations are well tolerated by higher species including humans. It is therefore possible that drug metabolism may be relevant to the differences in efficacy of SPI-77 in animal xenografts compared to advanced NSCLC.

An alternative hypothesis for the lack of clear correlation between preclinical and clinical efficacy of SPI-77 may relate to the differences in tumours. The size, location and vascularity of the tumours differ between xenograft tumours in mice and human tumours. Differences in the degree of vascularity and vessel permeability may lead to altered rates of liposomal diffusion into the tumour interstitium. It is worth noting that to date the most sensitive and attractive disease setting for STEALTH® liposomal doxorubicin is the setting of Kaposi's sarcoma where high vessel density and permeability are features. One might therefore theorise that SPI-77 may also be more active in settings where high vascularity and permeability are present.

The demonstrated activity of SPI-77 in human tumour xenografts does not completely exclude an inherent problem of the formulation of the cisplatin. The markedly protracted plasma kinetics may mean that free drug delivery to tumours takes place at a slow rate, thereby allowing adducts to be more successfully repaired. Certainly, the low levels of ultrafiltrated Pt are suggestive of retention of cisplatin by the liposomes, and it may be that the liposomal encapsulation is too retentive a vehicle to allow release of free drug. In the phase I study, two patients were assessed for tumour levels of Pt-DNA adducts and these were found to be low. This again could reflect either poor tumour penetration by the liposomes or inadequate release of free drug (Meerum Terwogt *et al*, 2002).

Although SPI-77 did not demonstrate sufficient activity to merit further study in advanced NSCLC, the trial demonstrated that large doses of liposomal cisplatin can be administered safely and with little toxicity. If an improved formulation of liposomal cisplatin can enhance drug delivery to the site of the tumour, then an agent with good activity and minimal toxicity might be developed for clinical use. Effective tumour targeting may optimise the radiosensitisation effects in combined therapy protocols in advanced NSCLC. Its application may also be extended to other cancers that are responsive to cisplatin, such as ovarian, head and neck and oesophageal cancer.

## Figures and Tables

**Table 1:** Patient's characteristics (*n*=26)

	**No.**	**%**
*Total*	26	
		
*Sex*		
Male	15	57.7
Female	11	42.3
		
*Age (years)*		
Median	61.5	
Range	45–76	
		
*Histology*		
Adeno	6	23
Squamous	14	54
Large	3	12
Other	3	12
		
*Performance status (ECOG)*		
0	0	0
1	19	73.1
2	7	26.9
		
*Disease stage*		
IIIA	2	7.7
IIIB	15	57.7
IV	9	34.6

ECOG=Eastern Oncology Cooperative Group.

**Table 2:** Toxicity (haematological and nonhaematological) (*n*=26)

**Toxicity**	**No.**	**%**
*Thrombocytopenia*		
Grade 1/2	—	—
Grade 3/4	—	—
		
*Neutropenia*		
Grade 1/2	1	3.8
Grade 3/4	—	—
		
*Anaemia*		
Grade 1/2	21	81
Grade 3/4	—	—
		
*Nausea*		
Grade 1/2	10	38
Grade 3	2	7.7
		
*Vomiting*		
Grade 1/2	5	19.2
Grade 3	2	7.7
		
*Itch*		
Grade 1/2	3	11.5
Grade 3	1	3.8
		
*Febrile reaction*		
Grade 1	2	7.7
		
*Rash*		
Grade 1/2	4	15.3
		
*Taste*		
Grade 1/2	3	11.5
		
*Tinnitus*		
Grade 1/2	1	3.8

**Table 3:** Pharmacokinetic parameters of patients treated with 100–260 mg m^−2^ SPI-77 i.v. during days 1–22

	**AUC_1–22_ (h *μ*g ml^−1^)**		**Clearance (l h^−1^)**		***V*_d_ (l)**		**Half-life (h)**	
**Dose SPI-77 (mg m^−2^)**	**Mean**	**s.d.**	**Mean**	**s.d.**	**Mean**	**s.d.**	**Mean**	**s.d.**
100	8233	2369	0.024	0.01	3.21	1.44	99.28	19.44
200	198 22	6186	0.019	0.002	3.00	0.7	120.83	32.31
260	227 29	4013	0.022	0.004	3.2	0.06	103.95	17.04

AUC=area under the curve; i.v.=intravenous; s.d.=standard deviation; V_d_=volume of distribution.
